# Microsurgical Management of Anterior Inferior Cerebellar Artery Aneurysms: Case Series and Review of Advanced Imaging and Cranial Base Approaches

**DOI:** 10.2174/0115734056399316251024065353

**Published:** 2025-11-05

**Authors:** Alejandro Serrano-Rubio, Julian-Moises Enriquez-Alvarez, Ambar-Elizabeth Riley-Moguel, Sharon-Paola Garcia-Trujillo, Brenda-Susana Hernandez-Barrera, Rafael Sanchez-Mata, Daniel Figueroa-Zelaya, Ernesto Roldan-Valadez, Edgar Nathal

**Affiliations:** 1 Department of Vascular Neurosurgery, National Institute of Neurology and Neurosurgery “Manuel Velasco Suárez”, 14267, Mexico City, Mexico; 2 Division of Research, Instituto Nacional de Rehabilitación “Luis Guillermo Ibarra Ibarra”, 14389, Mexico City, Mexico; 3 I.M. Sechenov First Moscow State Medical University (Sechenov University), Radiology Department, 119992, Moscow, Russia

**Keywords:** Basilar artery aneurysms, Fusiform aneurysms, Saccular aneurysms, Cerebral angiography, Microsurgery, AICA

## Abstract

**Background:**

Anterior Inferior Cerebellar Artery (AICA) aneurysms are rare, accounting for 0.1% to 0.5% of posterior circulation aneurysms. They often present with diverse morphologies and clinical symptoms, challenging diagnosis and management.

**Case Descriptions:**

We report three cases of AICA aneurysms with distinct clinical presentations and management strategies.

**Case 1:**

A 56-year-old male presented with chronic headache and left hemiparesis. MRI and 3D TOF MRA revealed a fusiform AICA aneurysm compressing the pons, treated with microsurgical clipping *via* anterior petrosectomy, resulting in a favorable outcome (mRS score of 0).

**Case 2:**

A 26-year-old female with a sudden-onset sentinel headache had a wide-neck saccular aneurysm of the right AICA confirmed by DSA. A posterior petrosectomy approach with clipping was performed, achieving complete aneurysm exclusion without complications (mRS score of 0).

**Case 3:**

A 21-year-old male with an incidentally detected saccular aneurysm underwent DSA and 3D angio-CT, confirming a wide-neck saccular aneurysm in the AICA territory. Microsurgical clipping *via* anterior petrosectomy was successful, with no residual lesion (mRS score of 0).

**Conclusion:**

Microsurgical clipping remains a viable option for managing wide-neck and fusiform AICA aneurysms, particularly those unsuitable for endovascular techniques. Advanced imaging modalities and tailored cranial base approaches are crucial for optimizing outcomes. Further studies are needed to refine management strategies for these rare aneurysms.

## 1 INTRODUCTION

The Anterior Inferior Cerebellar Artery (AICA) typically originates from the lower third of the basilar artery in about 75% of cases, the mid-third in 16%, and near the vertebrobasilar junction in 9% [1]. In most cases, the AICA forms a single vessel (72%), although duplication (26%) or triplication (2%) can occur. Anatomically, the AICA is divided into four segments: the anterior pontine segment, lateral pontine segment, flocculopeduncular segment, and segmental branches. These divisions are critical for understanding both the vascular supply to the cerebellum and the potential locations of aneurysms [2].

AICA aneurysms are rare, representing only 0.1% to 0.5% of all posterior circulation aneurysms [3]. They are most frequently located distally and are typically saccular in morphology [4]. The pathophysiology of AICA aneurysms is primarily due to arterial wall degeneration, often associated with atherosclerotic processes. When AICA aneurysms rupture, they may lead to Subarachnoid Hemorrhage (SAH), a condition associated with significant morbidity and mortality [5, 6]. Several factors predispose individuals to aneurysm rupture, including age, systemic arterial hypertension, previous SAH history, aneurysm size and location, geographic factors, and gender, with a higher prevalence in women [4].

AICA aneurysms are classified as proximal (from the BA-AICA junction to the AICA bifurcation or a combined AICA-PICA origin, premeatal segment) and distal (from the end of the meatal loop to the distal AICA, postmeatal segment) with reference to the seventh and eighth cranial nerve complex [4, 7-9]. The management strategies for these aneurysms vary significantly based on their location and morphological characteristics [10]. Due to the rarity of AICA aneurysms, comprehensive data regarding their anatomical and radiological features is limited, making clinical decision-making challenging [11].

Imaging modalities such as Computed Tomography (CT), Computed Tomography Angiography (CTA) with three-dimensional (3D) reconstruction, and Magnetic Resonance Imaging (MRI) are instrumental in the evaluation of AICA aneurysms. Digital Subtraction Angiography (DSA) remains the gold standard for assessing these lesions, providing detailed visualization of the aneurysm’s anatomy and aiding in the selection of appropriate management strategies [12].

Management options for AICA aneurysms primarily include microsurgical clipping and endovascular treatment. The choice between these approaches depends on factors such as the aneurysm’s location, size, configuration, and the patient’s clinical condition [13]. Microsurgical management often involves complex cranial base approaches because of the intricate anatomy of the AICA and its relationships with surrounding neurovascular structures [14]. These approaches include the pterional, subtemporal/Kawase, retromastoid, far lateral, supra-infratentorial combined, infratentorial supracerebellar approaches, lateral suboccipital, transcondylar, transcondylar fossa (supracondylar transjugular tubercle), extreme-lateral transcondylar, and transpharyngeal–transclival [13]. Each approach must be carefully selected based on the aneurysm's anatomical features to optimize surgical outcomes. For aneurysms deep within the skull, such as basilar trunk, vertebrobasilar junction, and many PICA aneurysms, other factors, such as osseous relationships and individual anatomical nuances of the skull base, can be just as critical [13]. Given the limited literature on AICA aneurysms,further studies are essential to clarify the most effective management strategies and improve outcomes for patients presenting with these complex vascular lesions [15, 16].

## 2 CASE DESCRIPTIONS

### 2.1 Case 1

A 56-year-old male presented with chronic headache and left hemiparesis. Magnetic Resonance Imaging (MRI) revealed a hypointense lesion with a peripheral mural thrombus compressing the pons on axial T2 sequences (Fig. **1A**). The sagittal T1 sequence displayed a rounded hypointense lesion with a peripheral mural thrombus rostral to the pons (Fig. **1B**). Three-dimensional time-of-flight (3D TOF) magnetic resonance angiography (MRA) identified a fusiform aneurysm with small lobulations in the anterior inferior cerebellar artery (AICA) (Fig. **1C**). Intraoperatively, a fusiform AICA aneurysm in the basilar portion was confirmed. The aneurysm was treated with microsurgical clipping using a straight 11 mm clip at the aneurysm neck. The patient's postoperative status was favorable, with a modified Rankin Scale (mRS) score of 0 at discharge.

### 2.2 Case 2

A 26-year-old female was admitted to the emergency department with a sudden-onset sentinel headache. Upon examination, her modified Rankin Scale (mRS) score was 1, and the Hunt and Hess scale score was II. DSA demonstrated a saccular aneurysm with a wide neck and small lobulations originating from the right AICA (Fig. **2A**). Surgical planning for a posterior petrosectomy was performed to treat the aneurysm (Fig. **2B**). The aneurysm was managed *via* a posterior petrosectomy approach with microsurgical clipping using a straight 9 mm clip. Postoperative angio-CT with three-dimensional reconstruction showed adequate aneurysm clipping with no residual lesion or evidence of vasospasm (Fig. **2C**). The postoperative outcome was excellent, with an mRS score of 0.

### 2.3 Case 3

A 21-year-old male was referred to our institution following the incidental discovery of a saccular aneurysm during a pan-angiography performed at an outside facility. The aneurysm, located in the basilar portion of the AICA, had a neck measuring 2.3 mm, a height of 7 mm, and a diameter of 3.5 mm. Digital subtraction angiography (DSA) performed at our institution confirmed a wide-neck saccular aneurysm dependent on the AICA (Fig. **3A**). Angio-CT with three-dimensional reconstruction demonstrated the saccular aneurysm in the AICA territory (Fig. **3B**). A microsurgical approach *via* anterior petrosectomy was undertaken. Intraoperatively, a saccular aneurysm was clipped using a straight 12 mm clip and a 15 mm fenestrated clip at the aneurysm neck. Postoperative CTA with 3D reconstruction showed adequate clipping of the aneurysm with no residual lesion or evidence of vasospasm (Fig. **3C**). The patient’s postoperative status was favorable, with an mRS score of 0 at discharge.

## 3 DISCUSSION

### 3.1 Clinical Variability and Imaging Findings: A Case-by-Case Analysis

AICA aneurysms are rare, representing less than 1% to 1.5% of all intracranial aneurysms [17]. The three cases presented in this report highlight the clinical variability and diverse anatomical presentations of AICA aneurysms, as summarized in Table **1**.

- Case 1 involved a 56-year-old male with chronic headache and left hemiparesis. Imaging findings on MRI and 3D TOF MRA revealed a fusiform AICA aneurysm compressing the pons (Figs. **1A**-**1C**). The anterior petrosectomy approach allowed for optimal visualization and clipping of the aneurysm. The patient's favorable postoperative outcome (mRS score of 0) supports the role of microsurgical clipping for fusiform aneurysms with a compressive effect.
- Case 2 presented a 26-year-old female with a sudden-onset sentinel headache. DSA and 3D angio-CT identified a wide-neck saccular aneurysm of the right AICA (Figs. **2A**-**2C**). The choice of a posterior petrosectomy approach facilitated safe clipping, resulting in complete aneurysm exclusion without residual lesion or vasospasm. The excellent outcome (mRS score of 0) emphasizes the efficacy of cranial base approaches for wide-neck saccular aneurysms.
- Case 3 involved a 21-year-old male with an incidentally discovered saccular aneurysm. DSA and 3D angio-CT confirmed a wide-neck saccular aneurysm in the AICA territory (Figs. **3A**-**3C**). Microsurgical clipping with dual clips provided effective aneurysm exclusion, with no postoperative complications (mRS score of 0). This case highlights the importance of detailed imaging assessment and individualized surgical planning in managing incidentally detected aneurysms.

### 3.2 Role of Imaging Modalities in AICA Aneurysms

Advanced imaging techniques played a crucial role in the diagnosis and management of AICA aneurysms. DSA complemented by CTA and MRA allowed precise characterization of aneurysm morphology, neck configuration, and spatial relationships with adjacent neurovascular structures. In particular, the integration of 3D angio-CT (Figs. **1C**, **2C**, and **3C**) was instrumental in confirming the adequacy of aneurysm clipping postoperatively. The use of multimodal imaging facilitated a tailored surgical approach for each case, optimizing outcomes and minimizing complications.

The superiority of DSA in delineating aneurysm morphology and guiding surgical planning is well-documented [6]. Its role in conjunction with 3D imaging modalities was evident in the precise assessment of aneurysm neck and morphology, which was critical for deciding between microsurgical and endovascular approaches [6].

### 3.3 Comparative Analysis of Surgical Approaches

Microsurgical clipping remains a cornerstone for managing complex AICA aneurysms, particularly for lesions with wide necks or fusiform morphology that pose challenges for endovascular techniques. The decision to opt for endovascular treatment should be made by the neurosurgeon in cases where the aneurysm's characteristics in relation to the intricate anatomy pose a greater risk of comorbidity, given the vascular neurosurgeon's experience. The use of cranial base approaches, such as anterior and posterior petrosectomies, offered direct access to the aneurysms while reducing the risk of neurovascular injury [18]. Several authors have reported the use of the endoscopic Endonasal Transclival Approach (EET) for treating aneurysms, but it has not been used for aneurysms affecting the proximal segment of the AICA [19]. The EET approach allowed exposure of a similar number of brainstem-perforating branches originating from the anterior pontine segment of the AICA. No anatomic structures limited the application of the proximal clip during the EET approach [19]. Cadaveric studies conclude that it is feasible to treat aneurysms of the anterior pontine segment of the AICA through the EET approach [19].

The selection of the anterior petrosectomy approach for Cases 1 and 3 was justified by the need for extensive exposure to fusiform and wide-neck saccular aneurysms, which are less amenable to endovascular techniques [20].

The posterior petrosectomy approach in Case 2 offered a favorable trajectory for the right AICA aneurysm, facilitating effective clip placement.

The uniform postoperative outcomes (mRS score of 0 for all cases) underscore the safety and efficacy of these tailored microsurgical approaches. The successful exclusion of aneurysms in all three cases supports the role of microsurgical clipping as a reliable option for AICA aneurysms, particularly in cases where endovascular access is limited by aneurysm morphology.

### 3.4 Clinical Implications and Future Directions

The findings of this report underscore the importance of a multidisciplinary approach that integrates advanced imaging techniques with individualized surgical planning. The favorable outcomes achieved in these cases highlight the continued relevance of microsurgical techniques for AICA aneurysms, despite the growing preference for endovascular treatments.

Given the rarity of AICA aneurysms, further studies with larger cohorts are necessary to establish evidence-based guidelines for their management. Comparative studies assessing the long-term outcomes of microsurgical clipping *versus* endovascular treatment are needed to refine treatment algorithms.

The management of AICA aneurysms requires a precise understanding of anatomical and radiological features, a tailored surgical approach, and the integration of advanced imaging modalities. The relevance of this paper is that, given the limited literature on the surgical management of AICA aneurysms compared with other series, it provides a current overview of the various treatment options, with a detailed analysis of their diagnosis using various imaging modalities, as well as their clinical outcomes. The successful outcomes reported here suggest that microsurgical clipping remains a valid and effective option for managing complex AICA aneurysms, particularly those with challenging morphologies and anatomical locations.

## 4 LIMITATIONS OF THE STUDY

In this study, we report only three cases of AICA aneurysms treated surgically *via* skull base approaches as mentioned above. Further studies with larger cohorts and comparisons with other treatment modalities, such as endovascular or endoscopic techniques, are needed to establish evidence-based guidelines for their treatment and long-term clinical outcome.

## CONCLUSION

AICA aneurysms are rare and present significant diagnostic and therapeutic challenges due to their diverse morphologies and complex anatomical locations. The case-by-case analysis presented in this report highlights the variability in clinical presentation and emphasizes the importance of multimodal imaging and individualized management strategies for optimizing outcomes, as summarized in Table **1**.

Microsurgical clipping remains a reliable treatment option for wide-neck and fusiform AICA aneurysms that are less amenable to endovascular techniques. The use of cranial base approaches provided effective exposure and enabled precise clipping of the aneurysms, with excellent postoperative outcomes (mRS score of 0 in all cases). Given the rarity of AICA aneurysms, further studies with larger sample sizes are necessary to develop standardized treatment protocols and to directly compare the long-term outcomes of microsurgical *versus* endovascular approaches.

In conclusion, the successful management of the AICA aneurysms presented here demonstrates that microsurgical clipping, supported by precise imaging and appropriate cranial base approaches, can achieve favorable clinical outcomes. Ongoing case reports and larger studies are required to expand the evidence base and refine management strategies for these challenging vascular lesions.

## Figures and Tables

**Fig. (1):**
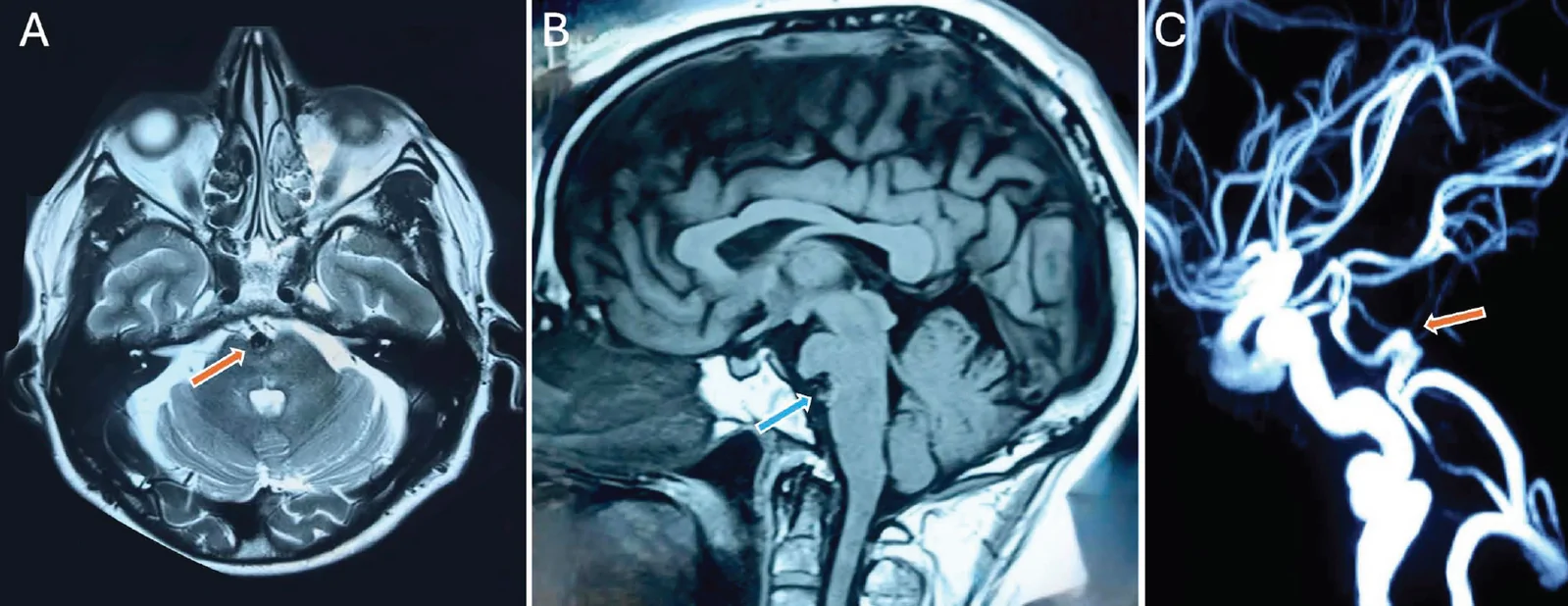
**A: **T2 axial sequence showing a predominantly hypointense rounded lesion with a peripheral mural thrombus compressing the pons (marked with the red arrow). **B: **T1 sagittal sequence displaying a rounded hypointense lesion with a peripheral wall thrombus adjacent to the pons (pointed with a blue arrow). **C: **Angio resonance 3D TOF demonstrating a fusiform aneurysm (marked with the red arrow) with small lobulations in the anterior inferior cerebellar artery (AICA).

**Fig. (2):**
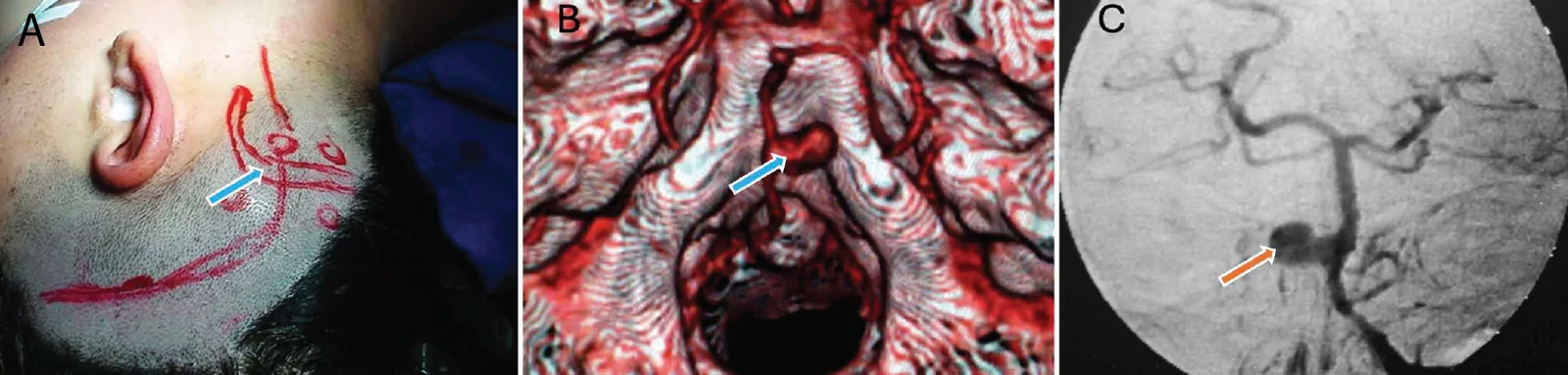
**A: **Surgical planning illustrating the posterior petrosectomy approach for treating a saccular AICA aneurysm (the blue arrow points to the sigmoid sinus). **B: **Preoperative angio-CT with three-dimensional reconstruction demonstrating a saccular aneurysm dependent on the right AICA (pointed with a blue arrow). **C: **Digital subtraction angiography (DSA) of the basilar artery showing a saccular aneurysm with a wide neck dependent on the right AICA (pointed with a red arrow).

**Fig. (3):**
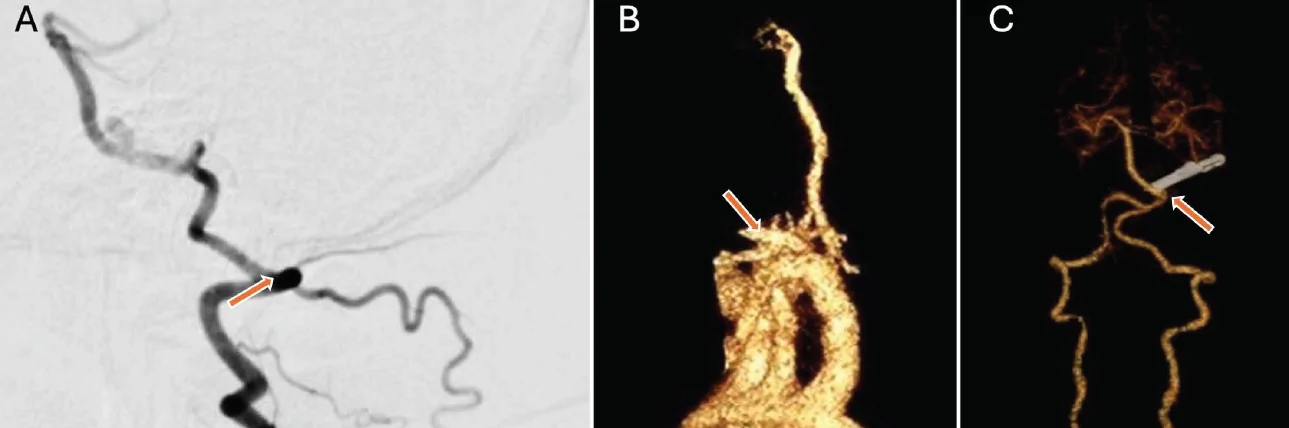
**A: ** Digital subtraction angiography (DSA) displaying a saccular aneurysm with a wide neck in the AICA territory (marked with the red arrow). **B: ** Angio-CT with three-dimensional reconstruction showing the saccular aneurysm in the AICA territory (pointed with a green arrow). **C: ** Postoperative angio-CT with three-dimensional reconstruction illustrating adequate clipping of the aneurysm without residual lesions or evidence of vasospasm (pointed with a red arrow).

**Table 1:** Summary of clinical and surgical features of patients with anterior inferior cerebellar artery aneurysms. This table presents the demographic characteristics, clinical presentation, imaging findings, aneurysm type and location, surgical approach, clipping technique, and postoperative outcomes for the three cases of anterior inferior cerebellar artery aneurysms.

**Feature**	**Case 1**	**Case 2**	**Case 3**
**Age / Gender**	56-year-old male	26-year-old female	21-year-old male
**Clinical Presentation**	Chronic headache, left hemiparesis	Sudden-onset sentinel headache	Incidental finding during pan-angiography
**Imaging Findings**	Hypointense lesion with peripheral mural thrombus (Figs. **1A**, **1B**); Fusiform aneurysm on 3D TOF MRA (Fig. **1C**)	Wide-neck saccular aneurysm on DSA (Fig. **2A**); Surgical planning for posterior petrosectomy (Fig. **2B**); Adequate clipping on 3D angio-CT (Fig. **2C**)	Wide-neck saccular aneurysm on DSA (Fig. **3A**); Aneurysm in AICA on 3D angio-CT (Fig. **3B**); Adequate clipping on 3D angio-CT (Fig. **3C**)
**Aneurysm Type and Location**	Fusiform AICA aneurysm in basilar portion	Saccular aneurysm in right AICA, basilar portion	Saccular aneurysm in AICA, basilar portion
**Surgical Approach**	Anterior petrosectomy	Posterior petrosectomy	Anterior petrosectomy
**Clipping Technique**	Straight 11 mm clip	Straight 9 mm clip	Straight 12 mm clip and 15 mm fenestrated clip
**Postoperative Outcome (mRS)**	0	0	0

## Data Availability

Alejandro Serrano-Rubio [A.S.R] had full access to all data in the study and takes responsibility for the integrity of the data and the accuracy of its contents.

## LIST OF ABBREVIATIONS

AICA: Anterior Inferior Cerebellar Artery
BA: Basilar Artery
CTA: Computed Tomography Angiography
DSA: Digital Subtraction Angiography
EET: Endoscopic Endonasal Transclival Approach
MRA: Magnetic Resonance Angiography
MRI: Magnetic Resonance Imaging
mRS: Modified Rankin Scale
SAH: Subarachnoid Hemorrhage
3D: Three-Dimensional
3D CTA: Three-Dimensional Computed Tomography Angiography
3D TOF: Three-Dimensional Time-of-Flight

## STANDARDS OF REPORTING

CARE guidelines were followed.
